# Factors influencing the detection of treatable epileptogenic lesions on MRI. A randomized prospective study

**DOI:** 10.1186/s42466-021-00142-z

**Published:** 2021-08-09

**Authors:** Tim Wehner, Philippe Weckesser, Steven Schulz, Annika Kowoll, Sebastian Fischer, Jessica Bosch, Leonie Weinhold, Rolf Fimmers, Matthias Schmid, Jörg Wellmer

**Affiliations:** 1 Ruhr - Epileptology, Department of Neurology, University Hospital Knappschafts-krankenhaus, Ruhr - University Bochum, In der Schornau 23 - 25, 44892 Bochum, Germany; 2 Department of Neuroradiology, University Hospital Knappschaftskrankenhaus, Ruhr - University Bochum, In der Schornau 23 - 25, 44892 Bochum, Germany; 3 Department of Medical Biometry, Informatics and Epidemiology, University Hospital Bonn, Venusberg Campus 1, Gebäude 11, 53127 Bonn, Germany

**Keywords:** Hippocampal sclerosis, Limbic encephalitis, Focal cortical dysplasia, Low grade epilepsy associated tumor, Nodular heterotopia, Gliotic scar

## Abstract

**Background:**

To prospectively analyze factors associated with detecting epileptogenic lesions on MRI within the work-sharing process of neurologists, epileptologists, radiologists and neuroradiologists.

**Methods:**

We assembled four sets of six MRI scans, each set representing five typical epileptogenic lesions (hippocampal sclerosis or limbic encephalitis; focal cortical dysplasias; periventricular nodular or other heterotopias; long-term epilepsy associated tumors; gliotic scar, hemosiderin or cavernoma), and non - lesional epilepsy.

At professional conferences, we invited neurologists, epileptologists, radiologists, and neuroradiologists to read two out of four MRI sets, one of which was presented with a clinical focus hypothesis. Participants were randomly assigned to MRI sets. Effects of examiners’ specialty, duration of training and professional experience on detection rate of epileptogenic lesions were investigated.

**Results:**

Fourty-eight neurologists, 22 epileptologists, 20 radiologists and 21 neuroradiologists read 1323 MRI scans. Overall, 613 of 1101 (55.7%) epileptogenic lesions were detected. Long-term epilepsy associated tumors (182/221, 82.4%) were found more frequently than gliotic scar, hemosiderin or cavernoma (157/220, 71.4%), hippocampal sclerosis or limbic encephalitis (141/220, 64.1%), nodular heterotopia (68/220, 30.9%) and focal cortical dysplasias (65/220, 29.5%, *p* < 0.001). Provision of a focus hypothesis improved the detection of hippocampal sclerosis or limbic encephalitis (86/110, 78.2% vs 55/110, 50%, p < 0.001) and focal cortical dysplasias (40/110, 36.4% vs 25/110, 22.7%, *p* = 0.037). Neuroradiologists and epileptologists were more likely than radiologists and neurologists to be amongst the most successful readers. In multivariable analysis, type of epileptogenic lesion, specialty of MRI reader, and provision of focus hypothesis predicted correct identification of epileptogenic lesions.

**Conclusions:**

Epileptogenic lesions are often not recognized on MRI even by expert readers. Their detection can be improved by providing a focus hypothesis. These results stress the need for training in the MRI characteristics of epilepsy - specific pathology, and, most importantly, interdisciplinary communication between neurologists/epileptologists and (neuro)radiologists to improve detection of epileptogenic lesions.

**Supplementary Information:**

The online version contains supplementary material available at 10.1186/s42466-021-00142-z.

## Introduction

Brain MRI is one of three cornerstones in the diagnostic workup of people with epilepsy, in addition to seizure semiology and interictal and ictal EEG. It allows diagnosing epilepsy after a first unprovoked seizure [1], and it is crucial to detect epileptogenic lesions (EL) potentially amenable to causative treatment options, such as immunomodulatory treatment in limbic encephalitis, or epilepsy surgery.

Yet published and everyday experience of epilepsy centers suggests that EL are frequently not identified at the time of the initial evaluation of the patient [2, 3].

Unlike EEG and video EEG, the acquisition and interpretation of MRI for neurological purposes is typically performed by radiologists (RAD) or neuroradiologists (NRAD) at the request of adult or pediatric neurologists (NEU) or epileptologists (EPI). This “dissociation” of treating and diagnosing physician may be justified by the expertise of the RAD and NRAD with regards to the respective examination technique. However, it may also be the cause of diagnostic failure. If relevant clinical information gets lost at the intersection between medical specialties, for example a clinical focus hypothesis (FH) on the most likely localization of the epileptogenic zone, the MRI reader might miss subtle lesions or over - interpret physiological variations as pathologic findings.

While it is accepted that providing a FH based on electroclinical findings to the MRI reader improves the detection rate of EL on MRI [3, 4], this issue has, to our knowledge, not been examined prospectively.

We therefore undertook this study to examine factors contributing to correct and incorrect reading of MRIs in patients with epilepsy. In addition to the role of a FH, we investigated how the MRI reader’s postgraduate education and experience with epilepsy - specific findings influenced the detection rates of ELs.

## Participants and methods

### Participants

Using a poster booth at international, national and regional medical conferences, annual meetings and summer schools (listed in the appendix), we invited adult and pediatric NEU, EPI certified by their International League Against Epilepsy (ILAE) chapter, RAD and NRAD to participate in the study.

In a questionnaire, participants were asked about their medical specialty, time since graduation from medical school, personal experience with creating a clinical FH in epilepsy based on seizure semiology and EEG, experience with reading MRIs of people with epilepsy, experience with reading MRIs at different magnetic field strengths, and training in epilepsy specific MRI.

### MRI - data

We assembled four sets (A, B, C, D) of each six MRIs of people with epilepsy. In each set one lesion of the subsequently specified five typical EL-categories (according to large patient series at tertiary epilepsy centers [5, 6]) and one non-lesional MRI were given. No individual lesion was represented more than once in each data set. The six lesion categories are:

- Hippocampal sclerosis or limbic encephalitis (HS/LE)
- Focal cortical dysplasia (FCD)
- Periventricular nodular or other heterotopia (PNH)
- Long-term epilepsy associated tumors (LEAT; ganglioglioma, dysembryoplastic neuroepithelial tumor, pleomorphic xantroastrocytoma)
- Gliotic scar / hemosiderin (e. g. due to cavernoma) (G/H/C)
- Non - lesional MRI (NL)

The lesions shown in the MRIs are representative for the common spectrum of the respective category in terms of their volume and signal characteristics. We did not stratify the lesions with regards to volume or signal specifics, neither within nor between lesion categories since this wouldn’t mirror clinical reality and was not the scope of this study. To find out, however, if lesion size correlates with their recognition we estimated the volume of each lesion (see below).

**Fig. 1:**
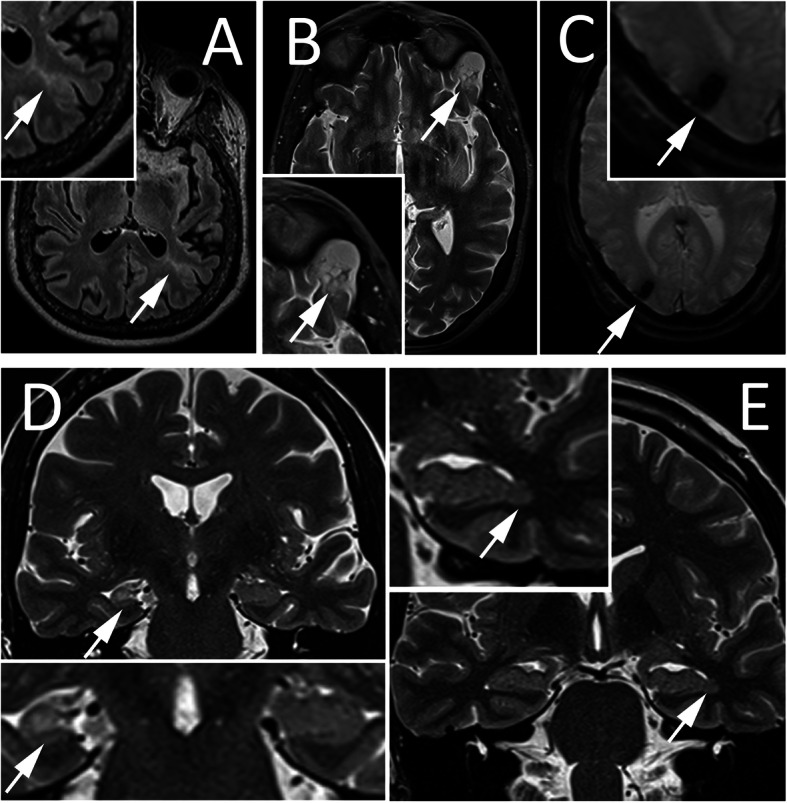
Representative images of epileptogenic lesions in MRI sets C. A Left frontal cortical dysplasia, axial FLAIR. B left frontal long-term epilepsy associated tumor, axial T2. C Right occipital hemosiderin deposits after hemorrhage, axial T2*. D right hippocampal sclerosis, coronal T2. E left periventricular nodular heterotopia, coronal T2

MRIs in 18 patients (including all in the FCD and NL groups) were acquired according to an epilepsy specific MRI protocol at our centre at a field strength of 3 T including the following sequences: volumetric MPRAGE (magnetization prepared rapid acquisition with gradient echo) 1x1x1mm, volumetric FLAIR (fluid attenuation inversion recovery) 1x1x1mm, T2 coronal 0.56 × 0.56 mm, 2.2 mm slice gap, T2 STIR axial images 0.45 × 0.45 mm, slice gap 3.75 mm, SWI axial 0.72 × 0.72 mm, 1.2 mm slice gap [7]. This protocol meets the HARNESS (harmonized neuroimaging of epilepsy structural sequences) criteria suggested by the ILAE neuroimaging taskforce on epilepsy [4]. In six patients (three in the G/H/C group, two LE, one PNH), examinations acquired at 1.5 T in outside practices were used, some lacking volumetric sequences. The EL, if present, was clearly recognizable in at least one sequence on the laptop computer used to display the MRI scans for the study. Lesion volume was estimated by the product of the maximal diameter in the axial, coronal and sagittal plane divided by 2. The lesions of MRI set C are shown in Fig. 1.

### Study design

In order to prevent potential bias from information exchange between study participants, each participant was asked to read two sets of six MRIs, one set provided with, the other without FH. Each lesion constellation was represented once in each set, hence each reader was asked to review two examples of each lesion constellation, once with, and once without FH. There are no established measures for difficulties of detection of individual lesions on MRI. Participants were therefore randomly assigned to the MRI sets stratified by their reported specialty.

EL entities were presented in a randomized order between the four MRI sets, MRIs with and without FH were presented to the participants in an alternating sequence:

- ABABABABABAB – MRI set A with FH
- BCBCBCBCBCBC – MRI set B with FH
- CDCDCDCDCDCD – MRI set C with FH
- DADADADADADA – MRI set D with FH

Time for review of the 12 MRIs was restricted to 60 min in order to mimic clinical practice settings. Participants documented their findings [EL yes/no; side; lobe; entity] on a protocol sheet and were asked to capture screenshots for verification of the found lesion. Set A and C contained one study, sets B and D two studies acquired at 1.5 T. Thus, each participant evaluated nine MRIs acquired at 3 T and three MRIs acquired at 1.5 T. This design with different MRI sets was chosen to minimize the risk of bias from potential information exchange between study participants.

Further methodological details and clinical information on the patients whose MRI scans were used are described in the additional information.

### Statistical evaluation

Participant characteristics are described using mean, median and quartile ranges for continuous variables and frequency distributions with percentages for categorical variables. Differences in participant characteristics between the different specialties were examined using Chi - Square, Kruskall - Wallis and ANOVA tests where applicable. Spearman’s correlation coefficient was calculated to examine the relationship between estimated lesion volume and detection rate.

For univariable and multivariable binary logistic regression analysis, correct identification of the EL on MRI [y/n] was used as outcome variable, and the following variables were used as covariates: lesion etiology [HS/LE; FCD; PNH; LEAT; G/H/C; NL], specialty of participant [NEU, EPI, RAD, NRAD], provision of FH [y/n], experience with reading MRI at different magnetic field strength [1, 1.5 T vs 3 T], completed training course on EL [y/n], experience in reading epilepsy MRIs [daily, weekly, monthly, yearly, none], experience in personally creating a FH [years], time since graduation from medical school [years].

Generalized linear mixed - effects models were used for univariable and multivariable binary logistic regression analysis. Since observations were not independent (physicians reviewed several MRIs each), physician ID was included in the analysis as a random factor to account for these dependencies. Additional univariable and multivariable mixed - effects models, with dependent and independent variables as described before, were fitted to the data stratified by lesion etiology. For multivariable analysis, all covariates with *p* < 0.2 in univariable analysis were included initially. Thereafter, the factor with the highest p - value was eliminated, until only factors with *p* < 0.05 remained. Effect sizes are reported as odd ratios compared to the value with the highest score within a given variable.

We used the software programs *IBM SPSS Statistics* (*version 23* and *24*) and *R (version 3.3.1.)* for the statistical evaluation.

## Results

### Participants

One hundred and twenty-five physicians consented to take part in the study, 111 (89%) read at least 10 MRIs and were included in the analysis. The only “dropout” reason for participants with < 10 MRIs readings was limited time in the conference setting. Descriptive statistics on participants are provided in Table 1. NEU had less postgraduate experience (quartiles 3.5, 9, 18.5y) than EPI (12, 19, 22y, *p* = 0.027), but not RAD (6.5, 14, 19.75y) and NRAD (10, 15, 22.5y). EPI (8, 10, 15y) had more experience with creating a FH than NEU (1, 2, 9y), NRAD (0, 0, 2y) and RAD (0, 0, 0y; *p* < 0.001). Allocation to MRI subsets was equally distributed across the four specialties (*p* = 0.99).

**Table 1:** Self - reported professional details of participants. Missing data were not provided by participants

MRI reader specialty		Neurology	Epileptology	Radiology	Neuroradiology	total
**Number of participants**		48	22	20	21	111
**Time since graduation from medical school**	< 5 y	12 (25%)	–	3 (15%)	2 (10%)	17 (15%)
	5–10 y	13 (27%)	4 (18%)	4 (20%)	4 (19%)	25 (23%)
	> 10 y	18 (38%)	17 (77%)	11 (55%)	13 (62%)	59 (53%)
	range, median	1–31, 9 y	6–34, 19 y	1.5–34, 14 y	2–35, 15 y	
	missing	5 (10%)	1 (5%)	2 (10%)	2 (10%)	10 (9%)
**Experience with creating a focus hypothesis in epilepsy (years)**	none	10 (21%)	–	17 (85%)	13 (62%)	40 (36%)
	< 5 y	19 (40%)	3 (14%)	–	3 (14%)	25 (23%)
	5–10 y	10 (21%)	10 (46%)	1 (5%)	4 (19%)	25 (23%)
	> 10 y	8 (17%)	9 (41%)	2 (10%)	1 (5%)	20 (18%)
	range, median	0–25, 2 y	2–30, 10 y	0–20, 0 y	0–15, 0 y	
	nissing	1 (2%)	–	–	–	1 (1%)
**Frequency of reading MRI in people with epilepsy**	never	11 (23%)	1 (5%)	2 (10%)	2 (10%)	16 (14%)
	yearly	13 (27%)	1 (5%)	7 (35%)	5 (24%)	26 (23%)
	monthly	16 (33%)	6 (27%)	8 (40%)	6 (29%)	36 (32%)
	weekly	5 (10%)	8 (36%)	3 (15%)	3 (14%)	19 (17%)
	daily	2 (4%)	6 (27%)	–	5 (24%)	13 (12%)
	missing	1 (2%)	–	–	–	1 (1%)
**Experience with maximum MR field strength**	1 Tesla	2 (4.2%)	–	–	–	2 (1.8%)
	1.5 Tesla	27 (56%)	–	7 (35%)	3 (14%)	37 (33%)
	3 Tesla	18 (38%)	22 (100%)	13 (65%)	18 (86%)	71 (64%)
	missing	1 (2%)	–	–	–	1 (1%)
**Training in epilepsy MRI**	yes	12 (25%)	10 (46%)	4 (20%)	7 (33%)	33 (30%)
	no	35 (73%)	12 (55%)	16 (80%)	14 (67%)	77 (69%)
	missing	1 (2%)	–	–	–	1 (1%)

### Results of MRI reading

A total of 1323 MRIs were evaluated, 761 (57.5%) were read correctly with respect to the study question (i. e. the EL was identified if present, or NL scans were read as containing no EL). The sensitivity to identify a given EL was 55.7% (613/1101), and 148/222 nonlesional scans (66.7%) were correctly identified as such. The detection rate of a given EL varied considerably across lesion entities and specialty of the MRI reader, and was better if a matching FH was provided, however provision of a FH did not result in more false-positive readings of nonlesional scans (Table 2). There was a moderate correlation (r = 0.544, *p* = 0.013) between lesion size and detection rate (Fig. S1, additional information).

**Table 2:** No of correctly read MRIs stratified by lesion category, provision of focus hypothesis, and reader specialty. *P*-values refer to the difference of detecting the lesion constellation with or without focus hypothesis for all readers

MRI reader speciality	Neurology		Epileptology		Radiology		Neuroradiology		total		p-value
**focus hypothesis provided**	**No**	**yes**	**No**	**yes**	**no**	**yes**	**no**	**yes**	**no**	**yes**	
**Focal cortical dysplasia**											
Correctly identified lesions/	7	16	9	9	3	6	6	9	**25**	**40**	**0.03**
Total scans	47	47	22	22	20	20	21	21	**110**	**110**	
	15%	34%	41%	41%	15%	30%	29%	43%	**22.7%**	**36.4%**	
**Hippocampal sclerosis or limbic encephalitis**											
Correctly identified lesions/	19	33	14	19	7	16	15	18	**55**	**86**	**< 0.001**
Total scans	47	48	22	22	20	20	21	20	**110**	**110**	
	40%	69%	64%	86%	35%	80%	71%	90%	**50.0%**	**78.2%**	
**Low grade epilepsy associated tumor**											
Correctly identified lesions/	34	36	20	15	18	19	19	21	**91**	**91**	**0.88**
Total scans	48	48	22	22	20	20	20	21	**110**	**111**	
	71%	75%	91%	68%	90%	95%	95%	100%	**82.7%**	**82.0%**	
**Nodular (periventricular) heterotopia**											
Correctly identified lesions/	6	9	9	10	7	5	11	11	**33**	**35**	**0.77**
Total scans	47	48	22	22	20	20	21	20	**110**	**110**	
	13%	19%	41%	45%	35%	25%	52%	55%	**30.0%**	**31.8%**	
**Gliotic scar or hemosiderin or cavernoma**											
Correctly identified lesions/	24	28	16	18	18	17	18	18	**76**	**81**	**0.46**
Total scans	48	47	22	22	20	20	20	21	**110**	**110**	
	50%	60%	73%	82%	90%	85%	90%	86%	**69.1%**	**73.7%**	
**Nonlesional**											
Correctly read/	25	37	18	14	15	10	13	16	**71**	**77**	**0.39**
Total scans	48	48	22	22	20	20	21	21	**111**	**111**	
	52%	77%	82%	64%	75%	50%	62%	76%	**64.0%**	**69.4%**	
**All scans**											
**Correctly interpreted/**	**115**	**159**	**86**	**85**	**68**	**73**	**82**	**93**	**351**	**410**	
**Total scans**	**285**	**286**	**132**	**132**	**120**	**120**	**124**	**124**	**661**	**662**	
	**40.4%**	**55.6%**	**65.2%**	**64.4%**	**56.7%**	**60.8%**	**66.1%**	**75.0%**	**53.1%**	**61.9%**	

Of the two samples for lesional categories presented to each participant, LEAT (182/221, 82.4%) were more likely to be identified than G/H/C (157/220, 71.4%), HS/LE (141/220, 64.1%), PNH (68/220, 30.9%), and FCD (65/220, 29.5%, *p* < 0.001). NRAD (175/248, 70.6%, range of EL 36–98%) were better than EPI (171/264, 64.8%, range 41–80%), followed by RAD (141/240, 58.8%, range 23–93%) and NEU (274/551, 48%, range 16–73%, *p* < 0.001). Provision of a FH overall improved the detection of an EL (333/551, 60.4% vs 280/550, 50.9%, *p* = 0.001). This effect was strongest in HS/LE (relative improvement of 56.4%, *p* < 0.001) and FCD (60%, *p* = 0.037), and modest or modestly negative for the other lesion entities. Readers with experience at reading MRI at 3 T were more successful than those without (532/847, 62.8% vs 220/464, 47.4%, p < 0.001), as were those who had completed a teaching course on EL (248/396, 62.6% vs 504/915, 55.1%, *p* = 0.011). Readers who evaluated MRIs in people with epilepsy daily (109/152, 71.7%) or weekly (145/227, 63.9%) were better than those who did this monthly (235/432, 54.4%), yearly (174/308, 56.5%), or less frequently (89/192, 46.4%, p < 0.001). Readers with > 10 years postgraduate experience identified more EL (448/707, 63.4%) than those with 5–10 years (155/294, 52.7%) or less than 5 years (100/202, 49.5%, p < 0.001) postgraduate experience. Experience in creating a FH in epilepsy for at least 5 years was associated with a better performance than no or < 5 years experience (329/539, 61.0% vs 423/772, 54.8%, *p* = 0.024). MRI field strength did not affect identification of a lesion constellation. Of HS/LE, 69/110 (63%) were identified at 1.5 T vs 72/110 (66%) at 3 T (*p* = 0.67), of PNH 21/56 (38%) at 1.5 T vs 47/164 (29%) at 3 T (*p* = 0.22), and of G/H/C 123/166 (74%) at 1.5 T vs 34/54 (63%) at 3 T (*p* = 0.12). Amongst the 74 false-positive readings, the most frequent specified false-positive reading was HS (*n* = 13, 18%), followed by FCD (*n* = 7, 9%), tumor (*n* = 5, 7%) and cyst (*n* = 4, 5%), however the majority were not specified (*n* = 36, 49%).

Since neither observations nor covariates were independent, we performed generalized linear mixed model analysis (Tables 3, 4). In univariable binary logistic regression analysis (Table 3), lesion etiology showed the strongest association with identification of EL. LEAT were twice as likely detected than G/H/C, almost 3x more readily found than HS/LE, and 13 - 14x more likely identified than PNH and FCD. NRAD and EPI were best at detecting EL, NRAD were 2.6x better than NEU and 1.7x better than RAD in identifying an EL. MRI Readers with > 10 years postgraduate experience identified 1.8x more EL than those with < 5 years experience. Detection of EL varied by a factor of 3 amongst those with daily or no MRI experience. Experience in reading MRI at 3 T vs 1.5 T (OR 2), having attended a course on MRI in epilepsy (OR 1.4), and provision of a FH (OR 1.4) also improved the detection rate of EL.

**Table 3:** Univariable binary logistic regression analysis, based on generalized linear mixed - effects model, entire dataset

Factors associated with correct interpretation of the MRI scan	p - value	Odds ratio	95% CI
***Lesion Category***			
Nonlesional	< 0.001	0.395	0.248–0.629
Hippocampal Sclerosis / Limbic Encephalitis	< 0.001	0.349	0.220–0.555
Periventricular Nodular Heterotopia	< 0.001	0.076	0.047–0.121
Gliotic scar / Hemosiderin / Cavernoma	0.005	0.505	0.315–0.810
Focal Cortical Dysplasia	< 0.001	0.070	0.044–0.113
Low Grade Epilepsy Associated Tumor	Reference		
***Medical Specialty***			
Radiology	0.04	0.590	0.354–0.985
Neurology	< 0.001	0.379	0.246–0.584
Epileptology	0.32	0.774	0.467–1.283
Neuroradiology	Reference		
***Postgraduate Experience***			
<5 y	0.01	0.550	0.342–0.884
5–10 y	0.03	0.634	0.420–0.958
≥10 y	Reference		
***Experience with MRI in Epilepsy***			
None	0.001	0.333	0.175–0.634
Yearly	0.03	0.509	0.282–0.918
Monthly	0.008	0.466	0.265–0.818
Weekly	0.28	0.707	0.378–1.326
Daily	Reference		
***Experience with MRI field strength***			
1 T	0.24	0.490	0.151–1.590
1.5 T	< 0.001	0.524	0.374–0.734
3 T	Reference		
***Experience with Focus Hypothesis in Epilepsy***			
None	0.64	0.891	0.551–1.443
<5 y	0.31	0.758	0.447–1.283
5–10 y	0.53	1.187	0.699–2.017
≥10 y	Reference		
***Teaching Course in MRI completed***			
No	0.07	0.716	0.497–1.032
Yes	Reference		
***Focus Hypothesis provided***			
No	0.001	0.682	0.544–0.855
Yes	Reference

**Table 4:** Multivariable binary logistic regression analysis based on general linear mixed - effects model, entire dataset

Factors associated with correct interpretation of the MRI scan	p - value	Odds ratio	95% CI
***Lesion category***			
Nonlesional	< 0.001	0.387	0.242–0.619
Hippocampal Sclerosis/Limbic Encephalitis	< 0.001	0.341	0.213–0.545
Periventricular Nodular Heterotopia	< 0.001	0.071	0.044–0.114
Gliotic scar / Hemosiderin / Cavernoma	0.004	0.498	0.308–0.803
Focal Cortical Dysplasia	< 0.001	0.065	0.040–0.106
Low Grade Epilepsy Associated Tumor	Reference		
***Medical Specialty***			
Radiology	0.04	0.512	0.270–0.972
Neurology	< 0.001	0.291	0.169–0.500
Epileptology	0.32	0.725	0.386–1.361
Neuroradiology	Reference		
***Focus Hypothesis provided***			
No	< 0.001	0.617	0.478–0.796
Yes	Reference		

After stepwise multivariate binary logistic regression, lesion etiology, medical specialty of the MRI reader, and provision of a FH were found to be the most important covariates in the prediction of correct scan reading (Table 4).

### Results stratified by lesion category

We then performed the same analysis separately for each lesion category (Tables S1, S2, additional information). NRAD were again 3.4–14.7 times better than NEU at detecting all EL except FCD. Regular experience in reading MRI and MRI experience at 3 T improved detection of all EL except for LEAT. Provision of a FH improved detection of HS/LE and FCD, but not the other types of EL.

In multivariate analysis (Table S2), regular experience and MRI experience at 3 T remained positive predictors for all EL except LEAT, and provision of a FH remained a predictor for detecting HS/LE and FCD.

### Profiles of good MRI readers

**Fig. 2:**
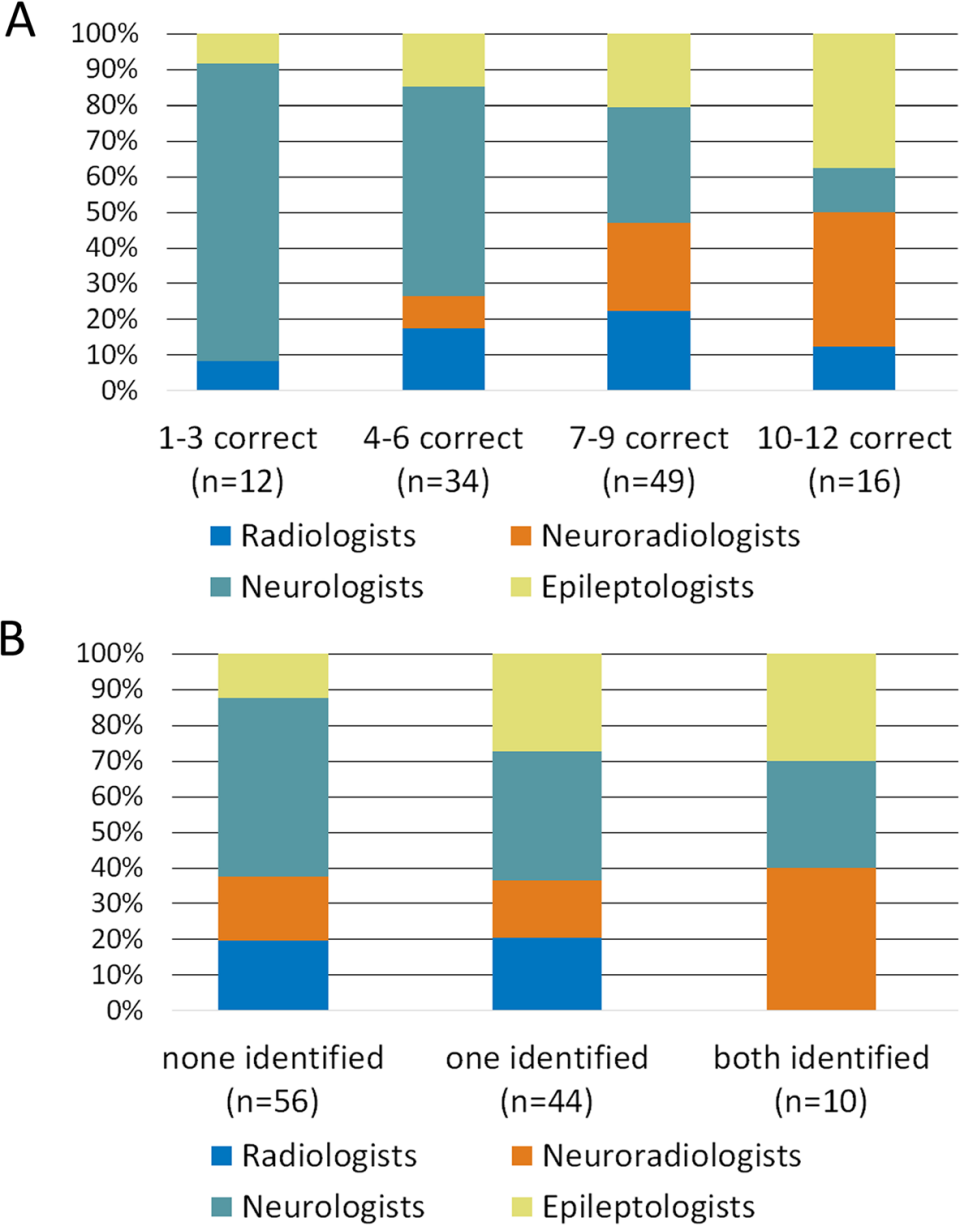
MRI - reading performance of medical specialties across all lesion entities (A), and focal cortical dysplasias only (B). Legend: A: Relative proportions of medical specialties among “poor” (1–3 correct diagnoses), “moderate” (4–6 correct diagnoses), “good” (7–9 correct diagnoses) and “very good” (10–12 correct diagnoses) MRI readers in percent across all MRIs presented. B: Relative proportions of medical specialties among 0, 1 and 2 correctly identified focal cortical dysplasias. One participant who reviewed only one MRI containing a focal cortical dysplasia was not included in this analysis

In addition to the analysis of individual variables contributing to the successful identification of EL, we examined the profiles of “very good” (10–12 correctly rated scans) to “poor” (0–3 correctly rated scans) MRI readers (Fig. 2A). Among the very good readers, there were more NRAD and EPI than RAD and NEU (*p* = 0.004, Chi square test). No RAD identified both FCD (Fig. 2B).

Irrespective of their medical specialty, the best readers (10–12 correctly read MRIs and two of two FCD detected) had > 10 years professional experience, including more than five years of experience with creating a FH, read epilepsy MRIs at least weekly, had experience with 3 T MRI, and had completed a course on epilepsy specific MRI.

## Discussion

There is ample literature that describes the effects of technical improvements in MRI on the delineation of EL (e. g. field strength of 3 T vs 1.5 T, slice thickness, planar angulation) [8–10], and epilepsy - specific MRI protocols have been proposed to transfer these improvements to patient care [4, 7, 10]. Far less literature has been published about factors on the side of the MRI reader that influence the detection of ELs on MRI.

In light of the fact that even at a quartiary epilepsy center, EL are often missed until re - review of MRIs in a presurgical conference, it is not surprising that MRI readers did not detect > 40% of EL in our study [11]. Yet, the detection of EL varied widely between different lesion entities.

LEAT and G/H/C were the types of EL that were most readily identified when no FH was provided, given that these EL usually have distinct signal alterations in T1 and T2 weighted sequences compared to both grey and white matter [8, 12]. Accordingly, these were correctly identified by most NRAD and RAD, i. e. clinicians one would expect to have acquired a well - calibrated ”set of eyes” for signal abnormalities, as well as a systematic approach to the analysis of MR images in different sequences and planes. Provision of a FH did not further improve the pick up rate for these lesion entities.

At the other end of the spectrum, only one third of PNH were identified, regardless of whether a FH was provided or not. PNH have the signal characteristics of grey matter [8, 12], and are therefore less obviously different from the surrounding brain tissue. This might also explain why provision of a FH did not improve the detection of PNH.

Conversely, HS/LE and FCD were more readily detected if a FH was provided. HS is characterized by hippocampal atrophy and loss of the internal architecture of the hippocampus (both seen on T2 - weighted images), and increased signal on T2 and FLAIR images [8, 10, 12]. MR features of LE are increased signal on T2 and FLAIR images and increased volume of the amygdala - hippocampal complex [13]. The detection of HS/LE therefore requires knowledge of these pathological findings, but seems to be facilitated if the FH directs the reader to the temporal lobes, a relatively circumscribed area for evaluation that can be easily compared to the contralateral side on each image.

Signal characteristics of FCD are subtle changes in gyral size and shape, decreased cortical T1 intensity, increased T2/FLAIR signal, and poor grey and white matter differentiation [8]. Focal cortical thickening can be difficult to identify since the cortical architecture in 2D images is prone to volume averaging, therefore the thickness of the cortical ribbon varies considerably even in an individual MR image.

In some cases, in particular in FCD type IIb, a “transmantle sign “of funnel - shaped high T2/FLAIR signal pointing towards the ventricle is seen [4, 12]. The tail of this hyperintensity is typically thin and may therefore be mistaken for an artefact or a perivascular space, or considered an unspecific white matter hyperintensity. Therefore, it is not surprising that FCD was the EL most often missed, but that its detection was facilitated if a FH was provided.

Nonlesional scans were correctly identified in about two thirds as such, i. e. participants incorrectly inferred an EL that was not present in about one third of cases, possibly because in the study setting they expected all MRIs to show an EL. Since we did not demand participants to provide a radiological diagnosis, we cannot draw further conclusions about the specifics of the misinterpretation of normal findings as EL. Failure to detect an EL has potentially far more reaching consequences for the patient than falsely diagnosing an EL that is not present, since one would expect the latter to trigger a referral to a subspecialist, in particular when it comes to consideration of surgical or immunomodulatory treatment.

Our finding that NRAD and EPI identified EL better than NEU and RAD parallels the longer postgraduate experience and longer training period, since NRAD and EPI are typically subspecialties that are open to clinicians with previous training in RAD and NEU, respectively. Postgraduate training is heterogeneously organized in the countries where we recruited our participants. We therefore relied on the self - reported (sub)specialty to categorize participants, rather than mandating certification by a particular professional board or organization. We did not ask for data on nationality or residency status, since these items were not relevant for our study.

Importantly, EPI identified more EL than RAD. This suggests that EPI via the regular exposure to epilepsy - specific pathologies (and probably also the widespread access of electronic MR images to EPI) can develop a competence in epilepsy - specific MRI that exceeds those of RAD. In professional settings where NRAD are not readily available, this competence should be utilized to improve the detection of EL in people with epilepsy.

Our study shows that detection of EL on MRI improves with the reader’s professional experience in epilepsy. It may be further improved by completing a course on MRI findings in epilepsy. Most importantly, sharing clinical information in the form of a FH with the MRI reader can increase the correct identification of an EL.

Based on our findings, we recommend that:

- MRIs of people with epilepsy should be read by clinicians with regular experience and specific training on MRI in epilepsy, regardless whether they have completed postgraduate radiological training.
- MRI readers should have access to a FH when reading the MRI. This calls for work - sharing in particular between general RAD and EPI or NEU.

Tele - Medicine solutions might improve the availability of expert MRI readers for a specific epilepsy - related question within a larger geographically area. It deservers further study to what extent our findings and interpretations may apply to other areas in neurology with work - sharing aspects.

Computer-assisted MRI quantification is used increasingly at tertiary epilepsy centers to assist in the detection of hippocampal pathologies, FCDs and heterotopias. However, these methods require technical expertise and a reference set typically acquired at the same scanner and are thus typically not available to the physicians performing the initial evaluation of the patient. Hence, we did not address the yield of these techniques in our study.

We acknowledge some limitations: Characteristics of study participants were self – reported, and some of the categories leave some room for discretion. This might have introduced bias affecting the results of the evaluation. On the other hand, it is likely that the recruitment process resulted in study participants with some degree of experience in epilepsy even amongst the non-specialists. We did not include board certification as a formal criterion to identify specialists, and no minimum requirements for MRI training courses were defined. Some lesion constellations (HS/LE, LEAT, G/H/C) contained different pathologies for different MRI sets / readers, as a compromise allowing inclusion of a broad spectrum of EL while acknowledging limited evaluation time for study participants. The MRI reading sessions were not done in a secluded office space with certified review conditions including state of the art equipment and optimized lightning used in neuroradiological practice, but for practical reasons on a laptop computer in a conference setting. This might have affected the performance of those readers used to review scans in optimal conditions. Less experienced MR readers might have performed better if the time for evaluation had not been limited to 60 min for 12 scans.

In order to limit bias from (potential) information exchange between study participants, we had to chose several scans for each lesion category. We are not aware of accepted criteria or a grading system for MRI difficulty. Therefore, we could only compensate for this by allocating participants to the MRI sets stratified by their reported specialty. MRI scans were chosen to represent the six most common lesion categories found in epilepsy surgery settings, which does not reflect a population based distribution of lesion categories.

## Conclusion

The detection of EL on MRI can be improved by providing the MRI reader with a FH and training in the MRI characteristics of epilepsy - specific pathology. Communication between neurologists/epileptologists and (neuro)radiologists is needed to improve detection of EL.

## Supplementary Information

**Additional file 1 **Methodological details and clinical information of the patients whose MRIs were used in the study. **Fig. S1.** Correlation of estimated lesion volume and detection rate. **Table S1.** Univariable binary logistic regression analysis based on generalized linear mixed - effects models, stratified by lesion category. **Table S2.** Multivariable binary regression analysis, based on generalized linear mixed - effects models, stratified by lesion category.

## Data Availability

The datasets used and/or analysed during the current study are available from the corresponding author on reasonable request.

## Abbreviations

EL: epileptogenic lesion
EPI: epileptologist
FCD: focal cortical dysplasia
FH: focus hypothesis
FLAIR: fluid attenuation inversion recovery
G/H/C: gliotic scar / hemosiderin (e. g. due to cavernoma)
HS: hippocampal sclerosis
ILAE: international league against epilepsy
LE: limbic encephalitis
LEAT: long-term epilepsy associated tumor
NEU: neurologist
NL: nonlesional
NRAD: neuroradiologist
PNH: periventricular or other heterotopia
RAD: radiologist
STIR: short tau inversion recovery
SWI: susceptibility weighted images
