# Supplementary material for: Factors influencing the detection of treatable epileptogenic lesions on MRI. A randomized prospective study
Source: Neurol Res Pract. 2021 Aug 9;3:41. doi: 10.1186/s42466-021-00142-z (PMC8351149; doi:10.1186/s42466-021-00142-z)
Supplement: Supplementary file 1 — Additional file 1 Methodological details and clinical information of the patients whose MRIs were used in the study. Fig. S1. Correlation of estimated lesion volume and detection rate. Table S1. Univariable binary logistic regression analysis based on generalized linear mixed - effects models, stratified by lesion category. Table S2. Multivariable binary regression analysis, based on generalized linear mixed - effects models, stratified by lesion category. [file 42466_2021_142_MOESM1_ESM.docx]

**Additional file for the manuscript**

**“Factors influencing the detection of treatable epileptogenic lesions on MRI. A randomized prospective study”**

1. **Methodological details and clinical information of the patients whose MRIs were used in the study** (873 words)
2. **Figure S1:** Correlation of estimated lesion volume and detection rate
3. **Table S1:** Results of univariable binary logistic regression analysis based on generalized linear mixed - effects models, stratified by lesion category
4. **Table S2:** Results of multivariable binary regression analysis, based on generalized linear mixed - effects models, stratified by lesion category.

Methodological details

Conferences where participants for the study were recruited comprised the annual meetings of the German Neurological Society 09/2014 (Munich, Germany), of the German Section of the ILAE 05/2014 (Bonn, Germany), of the Austrian Section of the ILAE 11/2014 (Linz, Austria), of the German Society of Neuroradiology 10/2014 (Cologne, Germany); as well as the conferences “Focus Neuroradiology (Fokus Neuroradiologie), 06/2014 (Hanover, Germany); “workshop on epilepsy” 01/2015 (Münster, Germany); and the Baltic Sea Summer School on Epilepsy 08/2014 (Trakai, Lithuania).

MRIs were displayed on laptop computers (screen size 15.6 inches, resolution 1366 x 768 pixels) shielded with poster walls to prevent daylight penetration in the conference setting. The laptops were equipped with an easy to use digital picture archiving and communication system (PerrPACS, written by Roy König, Bonn, Germany). This software allows display of multiple sequences including changing contrast and windowing simultaneously, as well as reformatting volumetrically acquired MRI sequences in the coronal, axial and sagittal plane, and labeling a location of interest in order to display it in a different sequence. The functionalities of PerrPACS were explained and technical assistance provided by two coauthors of the study (PW and SS) who were blinded to the content of the MRIs. Participants received a booklet containing a clinical FH on half of the patients whose MRIs they were asked to examine. The FH comprised a single cerebral lobe and side of the brain where an EL would be expected based on EEG findings and semiological seizure analysis. If provided, the FH was not misleading (i. e. the FH pointed to the lobe and side where the lesion was located, except for nonlesional scans). Four versions of this booklet allowed randomizing whether a specific MRI was presented with or without FH. Review conditions were identical for all participants.

Clinical information of the patients whose MRIs were chosen for the study

The age range of patients was 11 – 72, median 31 years. Four patients were <18 years old. Most patients had pharmacoresistant epilepsy and underwent presurgical evaluation where appropriate. Information presented here summarizes the conclusion of the evaluation; including results of histopathology were available.

The two patients with hippocampal sclerosis had temporal lobe epilepsy with seizure semiology and/or interictal and ictal epileptiform discharges pointing to the ipsilateral temporal region as the area of seizure origin. One patient underwent a temporal resection, histopathology confirmed hippocampal sclerosis. The two patients with limbic encephalitis had temporal lobe epilepsy with onset in adult life, memory loss, and clinical improvement with (partial) resolution of imaging findings following immunotherapy. No neuronal antibodies were found in CSF.

The four patients with focal cortical dysplasias all had MRI features suggestive of focal cortical dysplasias type II and focal onset seizures originating in the vicinity of the lesion based on semiology and/or EEG. In three patients, intracranial VEEG recordings showed typical signal characteristics of focal cortical dysplasia type II. These three patients became seizure free following stereotactically guided radiofrequency-thermocoagulation, a minimally invasive alternative to open resections that precludes histopathological examination of the coagulated tissue.^1^ In the fourth patient, surgical treatment was not entertained due to the large extent of the lesion in the left posterior quadrant.

Among the four patients with (periventricular) nodular heterotopias, one patient underwent noninvasive and subsequent intracranial VEEG suggesting seizure onset from the vicinity of the right frontopolar heterotopia, which was confirmed on histopathological examination. Another patient had focal onset aware seizures with vegetative symptoms (epigastric aura, nausea), followed in some by ictal speech and automatisms, head version to the left and bilateral tonic clonic generalization. He became seizure free following adjustment of his anti seizure medication. The third patient had pharmacoresistant epilepsy with bihemispheric seizure origins in the setting of bilateral nodular heterotopias. The fourth patient (Figure 1C) had focal onset aware and unaware seizures with oral automatisms, postictal aphasia, and/or bilateral tonic clonic evolution in some seizures. He declined surgical evaluation.

Three of the four patients with low grade epilepsy associated tumors underwent extended lesionectomies after noninvasive presurgical evaluation suggested seizure origin from the vicinity of the lesion. Histopathological diagnosis was ganglioglioma in two and pleomorphic xanthoastrocytoma in one. In the fourth patient, results of presurgical evaluation suggested seizure origin in the left mesial temporal area, which had normal appearances on MRI and FDG-PET, whereas the MRI lesion (Fig 1E) was found in the left lateral orbitofrontal area. The patient declined intracranial video EEG evaluation, the MRI lesion had stable appearance on three scans over a time period of three years.

The four patients in the G/H/C group had seizures with semiology and/or interictal EEG discharges suggesting seizure onset in the vicinity of the MRI lesion. One patient underwent resection, the histopathological diagnosis revealed hemosiderin deposits in the setting of teleangiectasic blood vessels.

Of the patients with nonlesional MRIs, one had focal onset aware seizures with cognitive and emotional symptoms and rare bilateral tonic clonic seizures, interictal EEG showed nonspecific right temporal slowing. One patient had reproducible right temporal sharp wave complexes on EEG with a clinical history of fasciculations but no seizures. One patient had bilateral tonic clonic seizures with unknown onset and normal EEGs. One patient was found to have multifocal seizure onset in the right hemisphere following intracranial EEG evaluation with strip and depth electrodes, precluding epilepsy surgery.

Reference:

1. Wellmer, J., Parpaley, Y., Rampp, S., Popkirov, S., Kugel, H., Aydin, Ü., Wolters, C.H., von Lehe, M., Voges, J.. (2016) Lesion guided stereotactic radiofrequency thermocoagulation for palliative, in selected cases curative epilepsy surgery. *Epilepsy Research, 121*(3):39‐46.

Figure S1: Correlation of estimated lesion volume and detection rate. There was a moderate correlation between estimated lesion volume and detection rate (r=0.544, p=0.013).


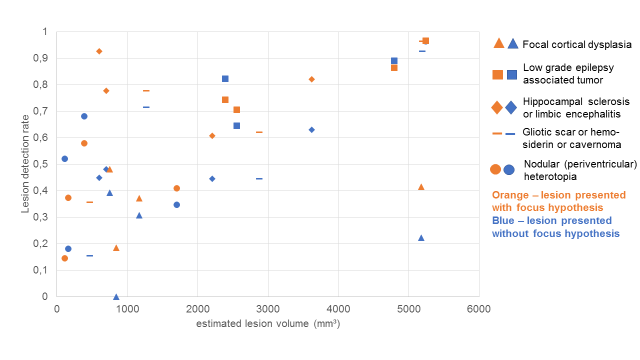


Table S1: Univariable binary logistic regression analysis based on generalized linear mixed - effects models, stratified by lesion category. Only factors significant at the 5% alpha level are shown. For nonlesional scans, no predictors were identified.

| **Factors associated with correct interpretation of the MRI scan** | **p - value** | **Odds ratio** | **95% CI** |
| --- | --- | --- | --- |
| **Hippocampal sclerosis / Limbic encephalitis** | | | |
| ***Medical Specialty*** | | | |
| Radiology | 0.04 | 0.328 | 0.112 - 0.956 |
| Neurology | 0.01 | 0.293 | 0.115 - 0.745 |
| Neuroradiology | Reference | | |
| ***Experience with MRI in Epilepsy*** | | | |
| None | 0.007 | 0.148 | 0.037 - 0.586 |
| Daily | Reference |  |  |
| ***Experience with MRI field strength*** | | | |
| 1.5 T | 0.006 | 0.404 | 0.212 - 0.770 |
| 3 T | Reference | | |
| ***Focus Hypothesis provided*** | | | |
| No | <0.001 | 0.266 | 0.146 - 0.486 |
| Yes | Reference | | |
| **Nodular (periventricular) heterotopia** | | | |
| ***Medical Specialty*** | | | |
| Neurology | <0.001 | 0.157 | 0.062 - 0.394 |
| Neuroradiology | Reference | | |
| ***Postgraduate Experience*** | | | |
| <5 y | 0.02 | 0.271 | 0.088 - 0.835 |
| ≥10 y | Reference | | |
| ***Experience with MRI in Epilepsy*** | | | |
| None | 0.003 | 0.116 | 0.029 - 0.475 |
| Yearly | 0.03 | 0.283 | 0.089 - 0.898 |
| Monthly | 0.002 | 0.166 | 0.054 - 0.514 |
| Daily | Reference | | |
| ***Experience with MRI field strength*** | | | |
| 1.5 T | <0.001 | 0.230 | 0.101 - 0.522 |
| 3 T | Reference | | |
| ***Experience with Focus Hypothesis in Epilepsy*** | | | |
| <5 y | 0.03 | 0.280 | 0.091 - 0.859 |
| ≥10 y | Reference | | |
| **Gliotic scar / Hemosiderin / Cavernoma** | | | |
| ***Medical Specialty*** | | | |
| Neurology | 0.001 | 0.168 | 0.059 - 0.476 |
| Neuroradiology | Reference | | |
| ***Experience with MRI field strength*** | | | |
| 1 T | 0.05 | 0.085 | 0.007 - 0.975 |
| 1.5 T | 0.003 | 0.370 | 0.191 - 0.716 |
| 3 T | Reference | | |
| ***Experience with Focus Hypothesis in Epilepsy*** | | | |
| None | 0.02 | 2.937 | 1.184 - 7.284 |
| ≥10 y | Reference | | |
| **Low Grade Epilepsy Associated Tumor** | | | |
| ***Medical Specialty*** | | | |
| Neurology | 0.01 | 0.068 | 0.009 - 0.538 |
| Epileptology | 0.04 | 0.098 | 0.011 - 0.849 |
| Neuroradiology | Reference | | |
| **Focal Cortical Dysplasia** | | | |
| ***Postgraduate Experience*** | | | |
| <5 y | 0.03 | 0.323 | 0.115 - 0.906 |
| ≥10 y | Reference | | |
| ***Experience with MRI in Epilepsy*** | | | |
| None | 0.007 | 0.205 | 0.065 - 0.649 |
| Yearly | 0.005 | 0.232 | 0.084 - 0.642 |
| Monthly | 0.001 | 0.193 | 0.073 - 0.509 |
| Daily | Reference | | |
| ***Experience with MRI field strength*** | | | |
| 1.5 T | 0.03 | 0.473 | 0.238 - 0.938 |
| 3 T | Reference | | |
| ***Experience with Focus Hypothesis in Epilepsy*** | | | |
| None | 0.02 | 0.381 | 0.169 - 0.859 |
| <5 y | 0.02 | 0.322 | 0.125 - 0.825 |
| ≥10 y | Reference | | |
| ***Teaching Course in MRI completed*** | | | |
| No | 0.005 | 0.406 | 0.218 - 0.755 |
| Yes | Reference | | |
| ***Focus Hypothesis provided*** | | | |
| No | 0.03 | 0.513 | 0.283 - 0.931 |
| Yes | Reference | | |

Table S2: Results of multivariable binary regression analysis, based on generalized linear mixed - effects models, stratified by lesion category. For Gliotic scar / Hemosiderin and Low grade epilepsy associated tumors, no further factors beyond the result of the univariable analysis were significant at the 5% alpha level.

| **Factors associated with correct interpretation of the MRI scan** | **p - value** | **Odds ratio** | **95% CI** |
| --- | --- | --- | --- |
| **Hippocampal sclerosis / Limbic encephalitis** | | | |
| ***Experience with MRI in Epilepsy*** | | | |
| None | 0.03 | 0.162 | 0.033 - 0.793 |
| Daily | Reference | | |
| ***Experience with MRI field strength*** | | | |
| 1.5 T | 0.02 | 0.388 | 0.177 - 0.853 |
| 3 T | Reference | | |
| ***Focus Hypothesis provided*** | | | |
| No | <0.001 | 0.238 | 0.125 - 0.453 |
| Yes | Reference | | |
| **Nodular (Periventricular) Heterotopia** | | | |
| ***Medical Specialty*** | | | |
| Neurology | 0.007 | 0.237 | 0.083 - 0.678 |
| Neuroradiology | Reference | | |
| ***Experience with MRI in Epilepsy*** | | | |
| None | 0.05 | 0.197 | 0.039 - 0.992 |
| Monthly | 0.02 | 0.243 | 0.072 - 0.817 |
| Daily | Reference | | |
| ***Experience with MRI field strength*** | | | |
| 1.5 T | 0.05 | 0.375 | 0.141 - 0.998 |
| 3 T | Reference | | |
| **Focal Cortical Dysplasia** | | | |
| ***Experience with MRI in Epilepsy*** | | | |
| None | 0.02 | 0.252 | 0.076 - 0.833 |
| Yearly | 0.005 | 0.221 | 0.077 - 0.636 |
| Monthly | 0.001 | 0.185 | 0.067 - 0.505 |
| Daily | Reference | | |
| ***Teaching Course in MRI completed*** | | | |
| No | 0.01 | 0.416 | 0.212 - 0.816 |
| Yes | Reference | | |
| ***Focus Hypothesis provided*** | | | |
| No | 0.02 | 0.468 | 0.247 - 0.888 |
| Yes | Reference | | |
